# HIV-related knowledge, information, and their contribution to stigmatization attitudes among females aged 15–24 years: regional disparities in Indonesia

**DOI:** 10.1186/s12889-022-13046-7

**Published:** 2022-04-01

**Authors:** Hidayat Arifin, Kusman Ibrahim, Laili Rahayuwati, Yusshy Kurnia Herliani, Yulia Kurniawati, Rifky Octavia Pradipta, Gevi Melliya Sari, Nai-Ying Ko, Bayu Satria Wiratama

**Affiliations:** 1 Department of Medical and Surgical Nursing, Faculty of Nursing, Universitas Padjadjaran, Jl. Raya Bandung, KM. 21, Jatinangor, Sumedang, 45363 West Java Indonesia; 2 Department of Community Health Nursing, Faculty of Nursing, Universitas Padjadjaran, Bandung, Indonesia; 3 Department of Medical and Surgical Nursing, Faculty of Nursing, Universitas Jember, Jember, Indonesia; 4 Department of Fundamental Nursing, Faculty of Nursing, Universitas Airlangga, Surabaya, Indonesia; 5 Department of Medical and Surgical Nursing, Stikes Husada Jombang, Jombang, Indonesia; 6 Department of Nursing, College of Medicine, National Cheng Kung University, Tainan, Taiwan; 7 Department of Biostatistics, Epidemiology, and Population Health, Faculty of Medicine, Public Health, and Nursing, Universitas Gadjah Mada, Yogyakarta, Indonesia

**Keywords:** HIV, Female, Knowledge, Stigma, Indonesia

## Abstract

**Background:**

Stigmatization attitudes among youths toward people living with HIV (PLWH) is still an issue and concern in Indonesia. The purpose of this study was to determine the regional disparities, levels of HIV-related knowledge, information, and contributions related to stigmatization attitudes among females aged 15–24 years in Indonesia.

**Methods:**

A cross-sectional study with The 2017 Indonesian Demographic Health Survey (IDHS) was used. A total of 12,691individual records of females aged 15–24 years were recruited through two-stage stratified cluster sampling. The endpoint was stigmatization attitude. Then, bivariate and multivariate binary logistics were performed.

**Results:**

The findings showed that female youths who have no HIV-related knowledge (62.15%) and some source of information (52.39%). The highest prevalence of stigmatizing attitude was 59.82%, on Java Island. Multivariate analysis showed that females living in Sulawesi and Kalimantan; those living in a rural area; and those with more HIV-related knowledge were less likely to have a stigmatizing attitude. Conversely, females with the middle- to richest-wealth index and had some HIV-related information were more likely to have a stigmatizing attitude.

**Conclusion:**

An understanding of stigmatizing attitudes should be considered through demographic factors, knowledge, and source of HIV-related information. The Indonesian government should pay more attention to indicators of HIV-related knowledge and information. Moreover, we suggest that the government collaborates with youths to disseminate information and restructure and reanalyze policies about HIV.

## Background

HIV (human immunodeficiency virus) is still a global problem and health burden [1]. At the end of 2019, it was estimated that worldwide, there were approximately 38.0 million people living with HIV (PLWH), of which 3.8 million were in Southeast Asia, including Indonesia [2]. According to the United Nations Programme on HIV/AIDS (UNAIDS) data, the number of PLWH in Indonesia has been increasing. From 2016 to 2018, the number of PLWH in Indonesia was 620,000, 630,000, and 640,000, respectively [3–5]. Based on age groups, the percentage of youths (15–24 years) living with HIV fluctuated from 2017 to 2019, at 21.1%, 18.1%, and 18.3%, respectively [6]. Based on regional reviews, the number of PLWH was uneven. There were regions with thousands of PLWH, while others had only ten PLWH [6]. The variations, instability, and inequality of the number of PLWH indicate a serious problem regarding regional disparities.

In addition to the increasing number of PLWH, another important problem related to HIV was stigmatization and discrimination aimed at PLWH, which increased from 57.1% in 2007 to 62.8% in 2012 [7]. Stigmatization is a social construction that devalues, labels, and connects the label against individuals or associated groups [8]. Moreover, discrimination occurs when the stigmatization is followed up in the form of actions or neglect of the stigmatized person. Discrimination is an act that refers to arbitrariness, neglect and restrictions [9]. Discrimination occurs at the interpersonal, community, and health service levels. At the interpersonal level, for example, stigmatization and discrimination from families result in isolation, differentiation in eating utensils [10] and ostracism [11]. At the community level, examples of stigmatization and discrimination include refusing to sit close to PLWH, changing seats away from PLWH, being afraid to physically interact with PLWH [10], dropping out of school, not being allowed to play together, and not eating with peers [11]. At the health services level, health care workers (HCWs) carry out stigmatization and discrimination by neglecting and not wanting to take care of patients in the hospital [10]. A further impact of discrimination on PLWH is the loss of jobs, social status, and support from family and the community; hence, they tend to avoid proper treatment [12]. Stigmatization and discrimination that result in reluctance to seek treatment for PLWH will worsen their health condition and increase their prevalence due to undetected spread.

To tackle HIV stigmatization and discrimination, the United Nations Programme on HIV and AIDS (UNAIDS) has developed a strategy adopted by the National AIDS Commission, termed getting to zero (zero infection, zero death, and zero discrimination) [13]. The Indonesian government also issued the Ministry of Health regulation number 21 of 2013 concerning the prevention of HIV and AIDS, and one of its goals was to eliminate discrimination against PLWH [14]. Unfortunately, even though strategies and regulations have been designed, several reports suggest that many people discriminate against PLWH. A total of 56% of household heads demonstrated high stigmatization to PLWH [15]. Nearly half (49.7%) showed a negative attitude toward people with HIV [16]. More than half (71.63%) of youths stigmatized PLWH [17], and younger people were 1.19 times more likely to discriminate against people with HIV [18].

Based on the data above, youths or younger people are more likely to discriminate against people with HIV. Youths do not understand and realize the forms of stigmatization and discrimination and their effects on people with HIV [19]. This is related to their young age, which is equally related to their lower cognitive, moral, and psychosocial development [20]. In addition, youths’ knowledge is related to their attitudes toward PLWH [21]. Youths with high and moderate knowledge have a positive attitude toward PLWH [22]. Youths are usually still in the era of ambivalence, which means that they still hold beliefs related to culture, norms, and religion and are also keeping up with technological advances, but have not completely dissociated from the influence of those beliefs, so the stigmatization of PLWH is still common [15].

Several previous studies in Indonesia on stigma toward PLWH have not shown a focus on youths. Previous studies have evaluated the determinants of stigmatization or discrimination but in respondents of all age levels [23], focused on PLWH’s parents [11], focused on youth respondents but did not describe the determinants of stigmatization attitudes [17], examined health care workers only [24], and only focused on ten regions [25]. In terms of measures to overcome stigma, around one-quarter of females did not give support for PLWH [26]. However, this study focuses on stigmatization by females and describes its determinants nationally. The purpose of this study is to reveal the determinants of female stigmatization toward PLWH. Given that females are more likely to demonstrate stigmatization, the results of this study are expected to be used for consideration and support to formulate policies to achieve zero stigmatization and discrimination.

## Methods

### Study design

We designed a cross-sectional study using secondary data from The 2017 Indonesian Demographic Health Survey (IDHS). The 2017 IDHS was nationally conducted by Statistics Indonesia, in collaboration with national agencies such as the National Population and Family Planning Board and the Ministry of Health of Indonesia. This survey was funded by the Indonesian government and took place from July 24 to September 30, 2017, in 34 provinces. The survey was technically assisted by Inner-City Fund (ICF) International, through The DHS Program [27].

### Samples

**Fig. 1:**
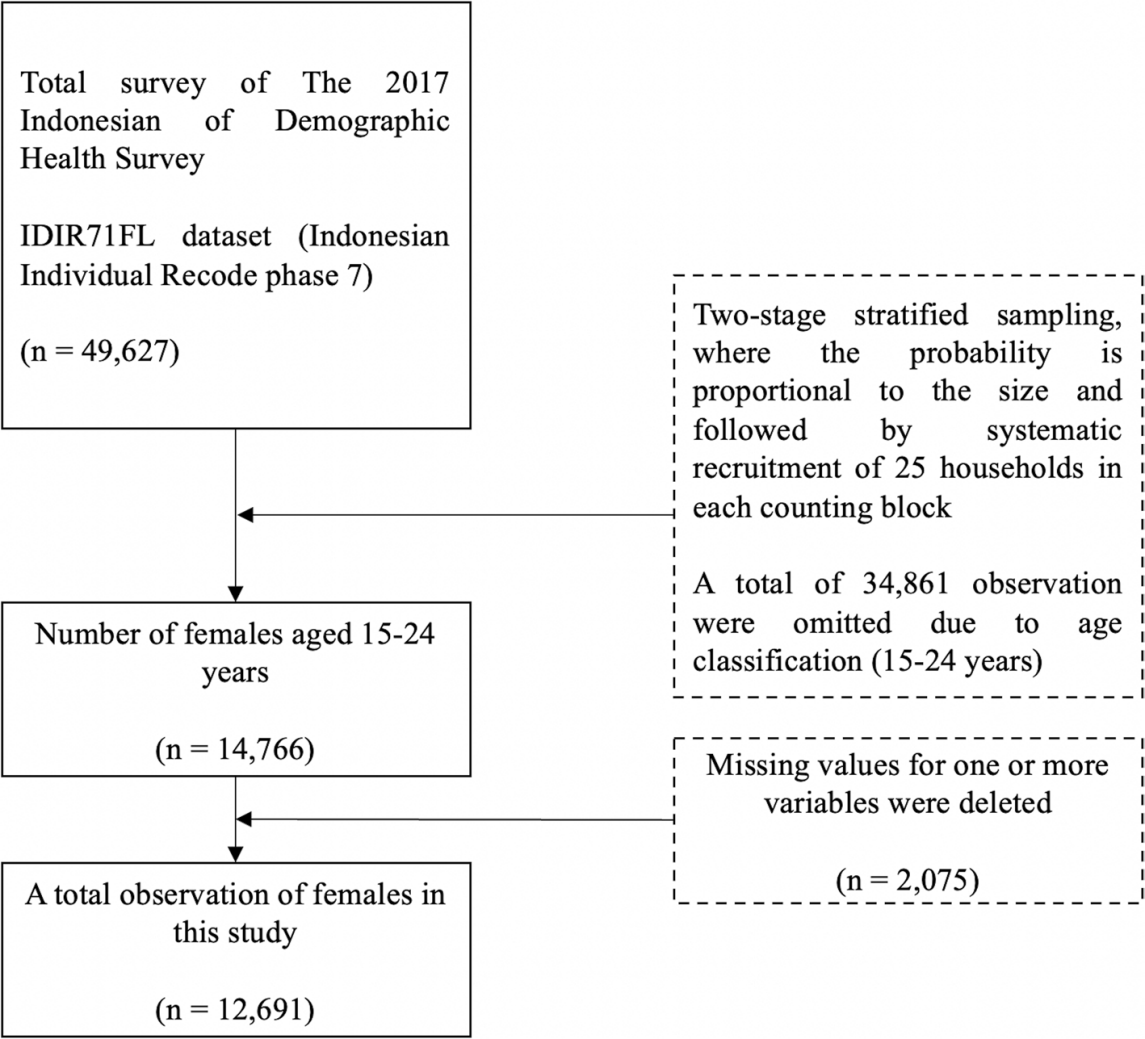
Flow chart for sample size selection

Samples were from The 2017 IDHS, which consists of 1,970 census blocks in urban and rural areas, with 49,250 household responses [27]. We used the IDIR71FL dataset (Indonesian Individual Recode phase 7). The total survey included 49,627 observations. The sampling design of the 2017 IDHS used two-stage stratified sampling, where the probability was proportional to the size, followed by the systematic recruitment of 25 households in each counting block [27, 28]. The inclusion criteria were youth aged 15–24 years. We excluded males aged 15–24 years because the prevalence of total survey from IDHS is less than three percent and based on the preliminary analysis, there were no significant relation in both genders. Thus, we only included females aged 15–24 years in the study. Furthermore, we weighted the observation based on the number of provinces in Indonesia to obtain the proportion of each region. Out of 49,627 records, approximately 34,861 observations were deleted due to limitations of age as only females (15–24 years) were required. Then, 2,075 records were rejected due to missing values for one or more variables. Finally, a total sample of 12,691 females was recorded in this study (Fig. 1).

### Variables

The independent variables in this study were region, age, sex, education level, marital status, wealth quintiles, residence, current work, HIV-related knowledge, and sources of information about HIV. Regions in Indonesia were categorized into Sumatera and Riau, Java, Bali and Nusa Tenggara, Kalimantan, Sulawesi, and Maluku and Papua. The categorization followed the method of a previous study [28]. Age and sex were classified into two categories (15–19 years and 20–24 years) and (male and female). Education levels were categorized as high, secondary, primary school, and no education [29]. Wealth quintiles were categorized as richest, richer, middle, poorer, and poorest [30, 31], which was determined based on principal component analysis (PCA) [32]. Furthermore, the place of residence was categorized as rural and urban [33]. The employment status of the respondent was determined based on the status of currently working (yes/no).

HIV-related knowledge variables were constructed from eight variables, namely, “Reduce risk of getting HIV: always use condoms during sex”, “Reduce risk of getting HIV: have 1 sex partner only, who has no other partners”, “Can get HIV by sharing food with person who has AIDS”, “HIV transmitted during pregnancy”, “HIV transmitted during delivery”, “HIV transmitted by breastfeeding”, “Can get HIV from mosquito bites”, and “A healthy looking person can have HIV.” Each variable was then recoded as “yes” and “no” to determine HIV-related knowledge [34]. A response of “do not know” was added to incorrect responses. The total score ranged from 0 to 8 and was classified into three categories: no knowledge (total score ≤ 50%; knowledge score 0–4), some knowledge (total score between 51%-74%; knowledge score 5–6), and more knowledge (total score ≥ 75%; knowledge score 7–8) as per a previous study [28]. Then, the Cronbach’s alpha was reported to be 0.60, indicating a reliable internal consistency.

Source of information about HIV was constructed from several variables, namely, “information from internet”, “newspapers”, “health professional”, “school”, and “television” [34]. Each variable was recoded with the statements “yes” or “no” to obtain a consistent measure of sources of information on HIV. The total score ranged from 0 to 5 and was classified into three categories: no information (total score ≤ 50%; score 0–2), some information (total score between 51%–74%; score 3), and more information (total score ≥ 75%; score 5), following the method of previous studies [28, 35]. We reported that the Cronbach’s alpha was 0.61, indicating a reliable internal consistency.

The dependent variable in this study was stigmatization attitude. This variable was constructed from five questions based on previous studies [36–38]. The questions related to stigmatization attitude included “would be ashamed if someone in the family had HIV”, “would want HIV infection in family to remain secret”, “people talk badly about people with or believed to have HIV”, “would be afraid to get HIV from contact with saliva from infected person”, and “people hesitate to take HIV test because of reaction of other people if positive” [34]. Each variable was then recoded as “yes” and “no” to determine HIV stigmatization attitude. A response of “do not know” was added to incorrect responses. The total score ranged from 0 to 5 and was classified into a dichotomized scale with the cut-off-point: no (total score ≤ 60%; score 0–3) and yes (total score > 60%; score 4–5). All responses were scored on a dichotomized scale (yes or no) based on previous studies [39, 40]. We reported that the Cronbach’s alpha was 0.70, indicating sufficient reliability for internal consistency.

### Data analysis

The STROBE statement was used as the standard for writing this study [41], and all of the methods used were performed in accordance with the relevant guidelines and regulations. The data analysis process was done by STATA version 16.1 software. We presented the weighted percentages of independent variables, dependent variables and regional distributions through univariate analysis. Furthermore, we examined the correlation of each variable using the chi-square test. Bivariate and multivariate binary logistic regression was used to analyze the factors related to stigmatization attitudes among youth. In the findings, we reported the adjusted odds ratio (aOR) along with a 95% confident interval (CI) and *p* value of < 0.05. We used “svy” survey commands in STATA to take into account the clustering effects and sampling weight due to multistage cluster random sampling used in the data collection since the national survey was used.

### Ethical consideration

Ethical consideration was performed and approved in Indonesia. We registered and requested access to the DHS dataset, received approval to access and downloaded the DHS data. The 2017 Indonesia Demographic and Health Survey was approved under the Institutional Review Board (IRB) Findings Form ICF IRB FWA00000845. Written informed consent for each individual was obtained by DHS. Information about ethical reviews is available on the website https://dhsprogram.com/Methodology/Protecting-the-Privacy-of-DHS-Survey-Respondents.cfm.

## Results

A total of 12,691 females aged 15–24 years old preceding the survey were interviewed. Table 1 shows that more than half of the respondents reported that they were on Java Island (59%). The highest proportion of females was in the age group of 15–19 years (52.17%). Approximately 74.12% had a secondary education level, and approximately 73.85% were unmarried. Regarding the wealth quintiles, the highest proportion was richest (23.71%), followed by richer (22.06%) and middle (21.27%), whereas only 13.34% belonged to the poorest household. More than half of the respondents lived in urban areas (56.3%), and most did not work (64.53%). From the study, more than half of females (53.95%) reported having stigmatization attitudes.

**Table 1:** Demographic variable (*n* = 12,691)

Variable	n	Weighted %
Regional (Island)		
Sumatera & Riau	3,268	20.61
Java	4,274	59
Bali & Nusa Tenggara	1,064	5.62
Kalimantan	1,043	5.21
Sulawesi	1,967	7.14
Maluku & Papua	1,075	2.42
Age		
15 – 19 years	6,749	52.17
20 – 24 years	5,942	47.83
Education Level		
High	3,101	21.17
Secondary	9,079	74.12
Primary	497	4.61
No	14	0.1
Marital Status		
Unmarried	9,700	73.85
Married	2,991	26.15
Wealth Quintiles		
Poorest	2,282	13.34
Poorer	2,504	19.61
Middle	2,535	21.27
Richer	2,615	22.06
Richest	2,755	23.71
Residence		
Urban	7,449	56.3
Rural	5,242	43.7
Currently Working		
No	8,327	64.53
Yes	4,354	35.47
Stigmatization Attitude		
No	6,005	46.05
Yes	6,686	53.95

Table 2 shows the indicator of HIV-related knowledge among female youths in Indonesia. Approximately 58.52% know about reducing the risk of contracting HIV by using condoms during sexual intercourse. A majority of females thought that HIV could be transmitted by sharing food (55.65%) or via mosquito bites (49.94%). Approximately 7.9% of females assumed that people infected with HIV were in an unhealthy condition. Overall, approximately 62.15% reported having no knowledge, while 36.87% having some HIV-related knowledge. Furthermore, from the source of information indicators, more than half reported obtaining information about HIV from school and watching television. Therefore, better dissemination of information from health professionals, newspapers, and the internet should be initiated. The proportions of female with no and some HIV information were 42.56% and 52.39%, respectively.

**Table 2:** Description of HIV-related knowledge and source of information among females aged 15–24 years in Indonesia (*n* = 12,691)

Indicators	Correct Answer	
	n	Weighted %
HIV-related knowledge		
1. “Reduce risk of getting HIV: always use condoms during sex”	7,182	58.52
2. “Reduce risk of getting HIV: have 1 sex partner only, who has no other partners”	10,151	81.26
3. “Can get HIV by sharing food with person who has aids”	7,121	55.65
4. “HIV transmitted during pregnancy”	10,401	83.14
5. “HIV transmitted during delivery”	9,176	73.16
6. “HIV transmitted by breastfeeding”	10,351	82.44
7. “Can get HIV from mosquito bites”	6,599	49.94
8. “A healthy-looking person can have HIV”	1,121	7.9
HIV-related knowledge		
No Knowledge	8,150	62.15
Some Knowledge	4,410	36.87
More Knowledge	131	0.98
Source of Information		
1. Information from internet	4,540	37.73
2. Information from newspaper	1,404	10.49
3. Information from health professional	2,007	13.84
4. Information from school	7,898	60.43
5. Information from television	6,842	53.91
Source of Information		
No Information	5,573	42.56
Some Information	6,494	52.39
More Information	624	5.05

Table 3 shows an overview of the regional disparities in Indonesia. More than half reported that they were females from Java Island (15–19 years vs. 20–24 years; 58.62% vs. 59.4%), whereas under 3% of females were from Maluku and Papua (15–19 years vs. 20–24 years; 2.52% vs. 2.3%). Moreover, the majority of females with a high education level lived on Java Island (58.49%). Conversely, in Maluku and Papua, 45.18% had no education, and most (60.43%) were married. The majority of the poorest- and richest-wealth indices were 33.15% and 70.84% on Java Island, respectively. The higher proportion of females who lived in urban and rural areas (67.37% vs. 48.21%) and who were currently working or not (59.62% vs. 58.65%) were on Java Island. A majority of female with more knowledge and some information about HIV were 67.47% and 61.97%, respectively, on Java Island. Most of the stigmatization attitudes were on Java Island (59.82%). Conversely, in Maluku and Papua, 2.76% did not have a stigmatization attitude.

**Table 3:** Socio-demographic, HIV-related knowledge, source of information, and stigmatization attitudes based on region in Indonesia (*n* = 12,691)

Variables	Sumatera & Riau	Java	Bali & Nusa Tenggara	Kalimantan	Sulawesi	Maluku & Papua
	**n (weighted %)**	**n (weighted %)**	**n (weighted %)**	**n (weighted %)**	**n (weighted %)**	**n (weighted %)**
Age						
15 – 19 years	1,683 (20.28)	2,223 (58.62)	594 (5.85)	554 (5.15)	1,096 (7.58)	599 (2.52)
20 – 24 years	1,585 (20.96)	2,051 (59.4)	470 (5.38)	489 (5.29)	871 (6.67)	476 (2.3)
Education Level						
High	822 (22.35)	866 (52.7)	276 (6.96)	228 (4.9)	601 (10.21)	308 (2.88)
Secondary	2,337 (20.27)	3,210 (60.61)	748 (5.31)	753 (5.16)	1,307 (6.45)	724 (2.2)
Primary	107 (18.08)	197 (62.8)	40 (4.64)	60 (7.39)	56 (4.14)	37 (2.94)
No	2 (13.66)	1 (18.84)	0	2 (13.67)	3 (8.64)	6 (45.18)
Marital Status						
Unmarried	2,570 (21.18)	3,224 (58.49)	825 (5.81)	758 (4.89)	1,504 (7.27)	819 (2.36)
Married	698 (18.98)	1,050 (60.43)	239 (5.11)	285 (6.14)	463 (6.78)	256 (2.57)
Wealth Quintiles						
Poorest	519 (26.13)	279 (33.15)	407 (12.3)	137 (6.88)	498 (12.82)	451 (8.71)
Poorer	695 (23.99)	661 (52.1)	226 (6.15)	229 (6.16)	451 (9)	242 (2.6)
Middle	730 (21.97)	896 (60.87)	133 (3.66)	265 (5.97)	340 (6.04)	171 (1.5)
Richer	700 (19.43)	1,103 (66.22)	136 (3.81)	217 (4.37)	329 (5.1)	130 (1.07)
Richest	624 (14.56)	1,335 (70.84)	162 (4.89)	195 (3.6)	364 (5.29)	75 (0.81)
Residence						
Urban	1,723 (16.19)	3,118 (67.37)	551 (5.3)	621 (4.32)	936 (5.24)	500 (1.58)
Rural	1,545 (26.29)	1,156 (48.21)	513 (6.04)	422 (6.37)	1,031 (9.6)	575 (3.49)
Currently Working						
No	2,084 (20.33)	2,682 (58.65)	742 (5.53)	633 (5.01)	1,390 (7.86)	796 (2.62)
Yes	1,184 (21.12)	1,592 (59.62)	322 (5.79)	410 (5.59)	577 (5.83)	279 (2.05)
HIV-related						
knowledge	2,361 (23.89)	2,484 (55.88)	574 (4.55)	692 (5.42)	1,342 (7.79)	697 (2.46)
No Knowledge			484 (7.52)	346 (4.94)	595 (5.99)	362 (2.33)
Some Knowledge	885 (15.2)	1,738 (64.02)	6 (2.45)	5 (2.32)	30 (9.34)	16 (3.31)
More Knowledge	22 (15.11)	52 (67.47)				
Source of Information						
No Information	1,450 (22.11)	1,674 (55.39)	68 (5.45)	498 (6.17)	884 (7.47)	599 (3.42)
Some Information	1,670 (19.69)	2,383 (61.97)	521 (5.47)	595 (4.47)	981 (6.7)	444 (1.69)
More Information	148 (17.37)	217 (58.48)	75 (8.67)	50 (4.92)	102 (8.99)	32 (1.57)
Stigmatization Attitudes						
No	1,384 (19.02)	1,934 (58.03)	499 (5.55)	529 (5.74)	1,125 (8.9)	534 (2.76)
Yes	1,884 (21.96)	2,340 (59.82)	565 (5.68)	514 (4.77)	842 (5.64)	541 (2.13)

Table 4 presents the results of bivariate analysis. Except for education level, marital status, and females working status, all the independent variables were associated with stigmatization attitudes (*p* < 0.05). Furthermore, the results of multivariate analysis are presented in Table 5. females who lived in Kalimantan and Sulawesi were 0.70 (0.61–0.81) and 0.56 (0.50–0.63) times, respectively, less likely to stigmatize. Moreover, we found that females within the richer wealth index were 1.24 (1.09–1.41) times more likely to stigmatize than those within the poorest wealth index. Females in rural areas had a 0.85 (0.79–0.92) times lower possibility of stigmatizing attitudes than those who lived in urban areas. Overall, youths with more HIV-related knowledge were 0.54 (0.38–0.78) times less likely to have stigmatizing attitudes than those with no knowledge of HIV. Conversely, youths with some information about HIV had 1.16 (1.07–1.25) times higher stigmatizing attitudes.

**Table 4:** Distribution of stigmatization attitudes by independent variables and bivariate analysis of factors association with stigmatization attitudes among females aged 15–24 years in Indonesia (*n* = 12,691)

Variables	Stigmatization Attitudes		X^2^	OR (95% CI)	*p*
	**No**	**Yes**			
	**n (weighted %)**	**n (weighted %)**			
Regional (Island)					
Sumatera & Riau	1,384 (42.5)	1,884 (57.5)	< 0.001	*Ref*	
Java	1,934 (45.3)	2,340 (54.7)		0.89 (0.79–0.99)	0.044
Bali & Nusa Tenggara	499 (45.48)	565 (54.52)		0.88 (0.75–1.01)	0.133
Kalimantan	529 (50.7)	514 (49.3)		0.71 (0.58–0.87)	0.001
Sulawesi	1,125 (57.4)	842 (42.6)		0.54 (0.47–0.63)	< 0.001
Maluku & Papua	534 (52.52)	541 (47.48)		0.66 (0.54–0.82)	< 0.001
Age					
15 – 19 years	3,254 (46.7)	3,495 (53.3)	0.031	*Ref*	
20 – 24 years	2,752 (45.34)	3,191 (54.66)		1.05 (0.96–1.15)	0.223
Education Level					
High	1,428 (44.73)	1,673 (55.27)	0.132	*Ref*	
Secondary	4,318 (46.09)	4,761 (53.91)		0.94 (0.85–1.05)	0.305
Primary	250 (50.73)	247 (49.27)		0.78 (0.62–0.98)	0.038
No	9 (78.94)	5 (21.06)		0.21 (0.05–0.78)	0.02
Marital Status					
Unmarried	4,590 (45.93)	5,110 (54.07)	0.992	*Ref*	
Married	1,415 (46.4)	1,576 (53.6)		0.98 (0.88–1.08)	0.709
Wealth Quintiles					
Poorest	1,212 (52.76)	1,070 (47.24)	< 0.001	*Ref*	
Poorer	1,208 (46.98)	1,296 (53.02)		1.26 (1.08–1.46)	0.003
Middle	1,188 (46.02)	1,347 (53.98)		1.31 (1.12–1.52)	0.001
Richer	1,153 (42.48)	1,462 (57.52)		1.51 (1.29–1.75)	< 0.001
Richest	1,244 (44.86)	1,511 (55.14)		1.37 (1.17–1.59)	< 0.001
Residence					
Urban	3,348 (43.81)	4,101 (56.19)	< 0.001	*Ref*	
Rural	2,657 (48.94)	2,585 (51.06)		0.81 (0.73–0.89)	< 0.001
Currently Working					
No	3,989 (46.71)	4,338 (53.29)	0.067	*Ref*	
Yes	2,016 (44.86)	2,348 (55.14)		1.07 (0.98–1.18)	0.11
HIV-related knowledge					
No Knowledge	3,887 (46.89)	4,263 (53.11)	0.001	*Ref*	
Some Knowledge	2,037 (44.36)	2,373 (55.64)		1.10 (1.00–1.21)	0.037
More Knowledge	81 (56.42)	50 (43.58)		0.68 (0.43–1.05)	0.087
Source of Information					
No Information	2,783 (48.6)	2,790 (51.4)	< 0.001	*Ref*	
Some Information	2,935 (44.03)	3,559 (55.97)		1.20 (1.09–1.32)	< 0.001
More Information	287 (45.49)	337 (54.51)		1.13 (0.92–1.38)	0.218

*X*^*2*^*: Chi-Square,CI: Confidence Interval,OR: Odds Ratio*

**Table 5:** Multivariate analysis of factors association with stigmatization attitudes among females aged 15–24 years in Indonesia (*n* = 12,691)

Variables	aOR (95% CI)	*p*
Regional (Island)		
Sumatera & Riau	*Ref*	
Java	0.83 (0.76–0.92)	< 0.001
Bali & Nusa Tenggara	0.86 (0.74–0.99)	0.045
Kalimantan	0.70 (0.61–0.81)	< 0.001
Sulawesi	0.56 (0.50–0.63)	< 0.001
Maluku & Papua	0.80 (0.69–0.92)	0.003
Age		
15 – 19 years	*Ref*	
20 – 24 years	1.03 (0.94–1.12)	0.497
Education Level		
High	*Ref*	
Secondary	0.99 (0.90–1.09)	0.924
Primary	0.95 (0.77–1.17)	0.678
No	0.48 (0.19–1.76)	0.343
Marital Status		
Unmarried	*Ref*	
Married	1.06 (0.95–1.17)	0.251
Wealth Quintiles		
Poorest	*Ref*	
Poorer	1.13 (1.01–1.28)	0.03
Middle	1.14 (1.01–1.29)	0.025
Richer	1.24 (1.09–1.41)	0.001
Richest	1.16 (1.02–1.33)	0.02
Residence		
Urban	*Ref*	
Rural	0.85 (0.79–0.92)	< 0.001
Currently Working		
No	*Ref*	
Yes	1.01 (0.94–1.10)	0.640
HIV-related knowledge		
No Knowledge	*Ref*	
Some Knowledge	1.01 (0.93–1.08)	0.823
More Knowledge	0.54 (0.38–0.78)	0.001
Source of Information		
No Information	*Ref*	
Some Information	1.16 (1.07–1.25)	< 0.001
More Information	1.12 (0.94–1.33)	0.171

*CI: Confidence Interval, aOR: adjusted Odds Ratio*

## Discussion

This study provides information about HIV-related knowledge and information, as well as factors that contribute to stigmatizing attitudes among female youths in Indonesia. Additionally, this study observed the regional distribution in Indonesia. Indonesia is an archipelagic country where development is still centered in western and central Indonesia. Thus, the distribution of the economy and education can be a concern. Indonesia consists of various cultures and beliefs that can have an impact on the perception and behavior of stigmatization attitudes. The majority of females are closely associated with the traditions and sociocultural that affect knowledge, perception, and belief [16]. The majority of Indonesians have the perception that HIV/AIDS is a disease from God because of someone’s behavior [42]. A related study showed that the society has a perception that PLWH are deviant people, such as commercial sex workers, drug abusers, and homosexuals [43].

In this study, it was found that females living in the eastern part of Indonesia, such as Kalimantan and Sulawesi, were less likely to exhibit stigmatizing attitudes than females living in other developed areas. In addition, in this study, it was also observed that females who live in rural areas were also less likely to exhibit stigmatization attitudes. The gaps were among people who live in urban areas, who were more likely to show stigmatization attitudes toward PLWH. This could be influenced by tolerance factors. Several previous studies have shown that people living in urban areas were more intolerant of PLWH [44–46]. Moreover, people who live in rural areas and far from urban areas had a higher level of tolerance and kinship and were willing to accept living together with PLWH [23, 37, 47, 48]. There was a lack of tolerance toward people with infectious diseases, thus encouraging stigmatization attitudes. Rural people know that HIV is not automatically transmitted through bad actions, such as free sex and drugs. A previous study showed that PLWH acquired through no risky behaviors experienced less discriminatory and stigmatizing attitudes [49].

Along with females living in urban areas, females within the middle to upper wealth index also tend to stigmatize. The gap between rich and poor people in HIV treatment needs to be reduced immediately because economic level is a key factor in determining the quality of life of an individual after a person is exposed to HIV. The stigma shown by female within the middle to upper economic status is due to an attitude that undermines PLWH. Most people believe that HIV at low economic status is associated with risky behavior [50] and sexual practices, such as having sex for money [51, 52]. However, the wealth index affects the ability to perform an HIV test. A previous study showed that wealthy female patients were more likely to report HIV testing in the past 12 months than were poorer patients [53]. This social gap may have an impact on stigmatization attitudes.

Media information is an important component in understanding HIV-related and stigmatization attitudes [54, 55]. However, there is much neglect related to media information about HIV, which reduces understanding and has an impact on stigmatization attitudes. This study found that respondents with some information had a greater tendency to stigmatize. The descriptive analysis showed that less than 60% of females did not receive any information about HIV from the internet, newspapers, or health professionals, and more than 35% did not receive information from school and television. These findings indicated that better utilization of information media and a greater role of health professionals are needed. In addition, factors related to stigmatization attitudes among people infected with HIV ignore the transmission mechanism and misunderstanding of the meaning of HIV itself, which causes fear that leads to rejection of PLWH [56]. A previous study on the stigmatization attitude of people showed a correlation with ignorance of information on how to transmit, prevent and treat HIV [43]. Strategies regarding health education for youths must be supported by providing education to community leaders or respected people in the area because they influence local cultural belief.

From the descriptive analysis of knowledge, we need to focus on how to increase females’ knowledge about HIV since more than 60% of them did not know about HIV. Specific knowledge about condom use, sharing food with PLWH, mosquito bites transmitting HIV, and attributing people who look unhealthy to those who have HIV should be a concern to the government and health professionals. However, we found that females who had more knowledge were less likely to have stigmatizing attitudes. A good level of education without being accompanied by proper understanding will engender negative attitudes [57]. A person’s good education level without being supplemented by an increase in individual awareness and acceptance of HIV-infected people will lead to negative attitudes [58]. A lack of HIV-related knowledge can lead to misconceptions, especially regarding disease transmission [59]. A previous study showed that there was a relationship between education and attitude whereby the higher the level of education a person had, the more positive was their attitude and acceptance of PLWH [43].

### Strengths and limitations

Our study provides information and findings about stigmatizing attitudes specifically held by females nationwide, which presents a regional distribution within Indonesia. We chose the youth age group because they often engage in stigmatization. For this reason, the results of this study illustrate that the right knowledge related to HIV should be given at an early age so that an attitude that leads to stigmatization does not occur in future. These results can be used as basic information for the government to determine further policies for reducing stigmatization attitudes among females toward PLWH. In this era of increasing globalization, governments are challenged with more responsibility. By having good knowledge about HIV, the government can collaborate with youths to disseminate proper understanding about HIV through social media campaigns and social acts to help reduce the stigmatization and discrimination attitudes. Moreover, youths have a greater responsibility to share with and have an influence on their peers regarding HIV through social media [60, 61].

However, the culture, beliefs, and religion of people in Indonesia were not evaluated by this survey. These variables would present more succinct information that could guide the approach of government in considering future policies.

## Conclusion

HIV-related knowledge and information are the basis for understanding how not to embrace stigmatizing attitudes. However, the findings showed that there were still many females who did not know about HIV and who lacked a health professional’s role in disseminating HIV-related information, which should also be a concern for the government. Moreover, there is a gap where females in areas far from the capital city of Indonesia have less of a stigmatizing attitude than those who live close to the capital city. In this study, it is also observed that stigmatization attitudes tended to be lower through better HIV-related knowledge. Furthermore, the government has an important role in preventing stigmatization attitudes toward PLWH by emphasizing correct information and facilitating understanding for youths within the territory of Indonesia through collaboration with health professionals and public figures.

## Data Availability

This study used data from Demographic and Health Surveys (DHS) for Indonesia, which are available from the DHS programme website (https://dhsprogram.com). The data set can be found in the following link- https://www.dhsprogram.com/data/dataset/Indonesia_Standard-DHS_2017.cfm?flag=1. The raw data of analysis during the current study are available from the corresponding author on reasonable request.
